# Tissue turnover of collagen type I, III and elastin is elevated in the PCLS model of IPF and can be restored back to vehicle levels using a phosphodiesterase inhibitor

**DOI:** 10.1186/s12931-016-0394-8

**Published:** 2016-07-05

**Authors:** Niels Ulrik Brandt Hansen, Morten Asser Karsdal, Sarah Brockbank, Simon Cruwys, Sarah Rønnow, Diana Julie Leeming

**Affiliations:** Nordic Bioscience A/S, Herlev Hovedgade 205-207, 2730 Herlev, Denmark; University of Southern Denmark, SDU, Odense, Denmark; Grünenthal Group, Aachen, Germany

**Keywords:** Precision-cut lung slices, *ex vivo*, IPF, Fibrosis, Biomarkers

## Abstract

**Background:**

The aim of this study was to develop and validate a model for pulmonary fibrosis, using *ex vivo* tissue cultures of lungs from bleomycin treated animals, enabling the investigation of fibrosis remodeling using novel biomarkers for the detection of ECM protein fragments. The combination of *in vivo* and *ex vivo* models together with ECM remodeling markers may provide a translational tool for screening of potential treatments for IPF.

**Methods:**

Twenty female Sprague-Dawley rats, twelve weeks of age, were administrated either two doses of bleomycin (BLM) (*n* = 14) or saline (*n* = 6) I.T., two days apart. Ten rats were euthanized at day seven and the remaining ten rats at day fourteen, after the last dose. Precision-cut lung slices (PCLS) were made and cultured for 48 h.

Ten female Sprague-Dawley rats, twelve weeks of age, were administrated either two doses of BLM (*n* = 7) or saline (*n* = 3) I.T., two days apart. The rats were euthanized fourteen days after the last dose. PCLS were made and cultured for 48 h in: medium, medium + 100 μM IBMX (PDE inhibitor), or medium + 10 μM GM6001 (MMP inhibitor).

Turnover of type I collagen (P1NP, C1M), type III collagen (iP3NP, C3M) and elastin degradation (ELM7) was measured in the supernatant of the cultured PCLS.

**Results:**

P1NP, C1M, iP3NP, C3M and ELM7 were significantly increased in supernatants from BLM animals (*P* ≤ 0.05 – *P* ≤ 0.0001) when compared to controls. P1NP, C1M, iP3NP, C3M and ELM7 were significantly increased in supernatants from day seven BLM animals compared to day fourteen BLM animals (*P* ≤ 0.05 – *P* ≤ 0.0001). P1NP, C1M, iP3NP, C3M and ELM7 were significantly decreased when adding IBMX to the culture medium of fibrotic lung tissue (*P* ≤ 0.05 – *P* ≤ 0.0001). C1M, C3M and ELM7 were significantly decreased when adding GM6001 to the culture medium (*P* ≤ 0.05 – *P* ≤ 0.0001).

Sirius Red and Orcein staining confirmed the presence of collagen and elastin deposition in the lungs of the animals receiving BLM.

**Conclusions:**

The protein fingerprint technology allows the assessment of ECM remodeling markers in the BLM PCLS model. By combining *in vivo*, *ex vivo* models and the protein fingerprint technology in the fibrotic phase of the model, we believe the chance of translation from animal model to human is markedly increased.

## Background

Idiopathic interstitial pneumonias are a diverse group of diseases characterized by fibrosis and inflammation, of which idiopathic pulmonary fibrosis (IPF) is the most common type. The etiology of the disease is unknown but it is believed to be an interplay between genetic predispositions and environmental causes, such as pollution and cigarette smoke [1]. The hallmark of fibroproliferative diseases can be described as a dysregulated wound healing, leading to a shift in the balance of extracellular matrix (ECM) remodeling and accumulation of matrix components and end stage fibrosis with loss of function of the affected organ. The total ECM turnover is increased in IPF, meaning that both formation and degradation of proteins is upregulated, but the net result is matrix deposition. The formation and degradation of matrix proteins generate protein fragments, which are released into circulation, forming neoepitopes that may be measured as biomarkers. A paper just published in The Lancet describes how novel biomarkers of the ECM were able to separate fast progressing IPF patients from stable IPF patients, while some of the markers were also strongly predictive of overall survival [2]. The ECM has gained a lot of attention during the last decades and it is now recognized as a structure with multiple functions such as cell support, cytokine activation and signaling purposes [3]. It is known to be important for the phenotype and survival of cells, e.g. orientation of collagen can critically regulate cell and tissue behavior [4–6]. Moreover the ECM can serve as a storage site for multiple soluble/secreted cytokines and growth factors, thereby functioning as a repository. These factors can be released into the microenvironment upon matrix degradation by matrix metalloproteinases (MMPs) [7, 8]. The accumulation of ECM proteins also affects the rigidity of the matrix causing the matrix to become stiff. This stiffness affects the communication and signaling of the matrix and hence results in the thickening of the interstitium in the alveoli, which leads to less O_2_ uptake and shortness of breath. The stiffness has also been shown to affect myofibroblast activation which ultimately leads to more matrix deposition and rigidity, thus creating a positive feedback loop and linking matrix architecture and composition with cell phenotype [9–11].

The precision cut lung slice model using IPF tissue enables the possibility of studying the fibrotic processes in a multicellular system in which cell-cell, cell-ECM interactions and opened alveoli are preserved, thus maintaining the 3D structure seen *in vivo* [12, 13]. The importance of the interplay between the cells and the microenvironment is very well described within the cancer field. Important factors such as the presence of all the different cell types, structural support from the ECM, signaling (e.g. via transmembrane receptors) and cytoskeletal and chromatin organization are some of the highlighted features. In order to get full insight into the biology of normal and diseased tissue, it is vital to create *in vivo*-like models for therapeutic testing to get the full context of the effects [14–17]. In addition, the PCLS model enables the study of a heterogeneous matrix, which provides an *in vivo*-like environment with minimum alterations of the natural conditions in order to study cell behavior and function. This is a very crucial matter when testing potential therapies [3]. Due to the importance and influence of the matrix in IPF, we used the fit for purpose Precision-Cut Lung Slice (PCLS) model [18] using *in vivo* BLM treated lung tissue combining it with a novel Protein Fingerprint biomarker technology for the assessment of the ECM remodeling.

We have chosen two compounds, IBMX (non-selective phosphodiesterase inhibitor) and GM6001 (pan MMP inhibitor), as they have been shown to have a positive effect on remodeling biomarkers and are non-toxic in *ex vivo* models of the liver and cartilage [12, 19, 20]. The treatments were applied in what is generally accepted as the fibrotic phase, at day 14 following BLM administration.

We combined the rodent PCLS model with *in vivo* BLM treated lung tissue and the novel Protein Fingerprint biomarker technology for the assessment of the ongoing ECM protein remodeling. The aims of this study were to design and validate a model for screening of potential therapies for IPF.

## Methods

### Reagents

All reagents were standard high-quality chemicals from either Merck or Sigma-Aldrich. The culture medium William’s Medium E was from Gibco (Life Technologies), while AlamarBlue assay was from AbD Serotec.

### Animal experiment

Thirty female Sprague-Dawley rats, aged twelve weeks, were used for two experiments and were housed at the animal facilities at Nordic Bioscience, Denmark. The experiments were approved by the Animal Ethics Committee of the Danish Ministry of Justice (2011/561-2003, 2012-15-2934-00467). The rats were housed in standard cages, with bedding and nest material at 18-22 °C and fed with standard pellet diet and tap water ad libitum. The rats were kept under conditions of a 12 h light/dark cycle. Pulmonary fibrosis was induced by I.T. installation of two doses of BLM (0.25 ml/kg), two days apart. The BLM was sprayed into the lungs of the rats using the MicroSprayer® Aerosolizer with the FMJ-250 High Pressure Syringe (Penn-Century, PA, USA). In the first experiment fourteen animals were given BLM and six animals were given saline. Ten animals were euthanized seven days after the last BLM/saline dose, and ten animals were euthanized fourteen days after the last BLM/saline dose.

In the second experiment seven animals were given BLM and three animals were given saline, two days apart. The animals were euthanized fourteen days after last BLM/saline dose.

### *Ex vivo* experiments

Prior to euthanization of the rats, the animals were anesthetized and a small catheter was surgically inserted into the trachea. After euthanization, the catheter was used to fill the lungs with a low temperature gelling agarose in order to keep the lungs dilated. The lungs were excised and stored in ice-cold Krebs-Henseleit buffer containing 25 mM glucose, 10 mM HEPES and 25 mM NaHCO_3_. PCLS were prepared from the lungs using a TSE Krumdieck tissue slicer MD 4000 as previously described [18]. The PCLS were cultured for 48 h, as longer incubation would affect cell viability, at 37 °C under carbogen atmosphere in sterile 48 well plates containing 300 μl William’s Medium E containing 25 mM glucose, 50 μg/mL gentamicin and the respective treatment. Treatments used for the *ex vivo* model were 100 μM IBMX (Sigma-Aldrich, Denmark), a non-selective PDE inhibitor, and 10 μM GM6001 (Sigma-Aldrich, Denmark), a pan MMP inhibitor. After the culturing period the supernatants were collected and stored at -20 °C until use.

### Cell viability measured by AlamarBlue

AlamarBlue is a sterile non-toxic aqueous oxidation-reduction indicator that yields colorimetric changes and a fluorescent signal in response to metabolic activity. The active compound, resazurin, is an oxidation-reduction indicator that changes from the oxidized non-fluorescent form to the reduced fluorescent form according to the viability and proliferating activity of cells [21]. In short, 300 μL 10 % AlamarBlue was added to each well of the microtiter plate containing PCLS and incubated for 90 min at 37 °C while shaking. The results were measured by an ELISA reader at 540-590 nm.

### ELISA measurements

The P1NP ELISA is specific for the N-terminal pro-peptide of type I collagen. It is a marker of collagen type I formation as the pro-peptide is cleaved off the mature protein prior to fibril assembly [22]. The C1M marker is specific for a fragment generated by MMP-2, -9 and -13, and hence reflects MMP generated degradation of collagen type I [23]. The iP3NP marker is specific for an internal epitope of the N-terminal pro-peptide of type III collagen, which reflects the formation of collagen type III (unpublished). The C3M marker is specific for the a neo-epitope generated by MMP-9, and reflects the degradation of type III collagen [24]. The ELM7 marker is specific to a neo-epitope generated by MMP-7 degradation of elastin [25]. All the above ELISA assays are developed and validated by Nordic Bioscience (see references for the technical papers). The markers were assessed in the supernatants of the cultured PCLS, i.e. the release of the epitopes into the supernatants.

### Histological analysis

Lung tissue were fixed in cold 4 % neutral formalin diluted in phosphate-buffered saline at 4 °C and processed for paraffin embedding using a Sakura Tissue Tek VIP 5 Jr. Tissue Processor and cut on a microtome in 5 μm-thick slides.

### Sirius Red

The slides were deparaffinized, rehydrated and incubated with Weigert’s Haematoxylin for 8 min, washed in water, and then incubated with a 0.1 % Sirius Red solution dissolved in aqueous saturated picric acid for 1 h, washed in water, dehydrated and mounted with PERTEX®. Pictures were taken with an Olympus BX60 microscope mounted with an Olympus DP71 camera at 4x magnification.

### Orcein stain

The slides were deparaffinized, rehydrated and incubated with Acid Orcein Solution for 30 min, washed in water, and then incubated with Mayer’s Haematoxylin Solution for 30 s, washed in water, dehydrated and mounted with PERTEX®. Pictures were taken with an Olympus BX60 microscope mounted with an Olympus DP71 camera at 40x magnification.

### Statistical analyses

Results are shown as mean ± standard error of mean (SEM). Differences between mean values were compared by nonparametric Mann-Whitney’s *t*-test for two-tailed observations. All statistical analyses were performed in GraphPad Prism software v.6 (GraphPad Software, San Diego, CA). P values less than 0.05 were considered significant.

## Results

### ECM remodeling is increased in fibrotic lung

**Fig. 1:**
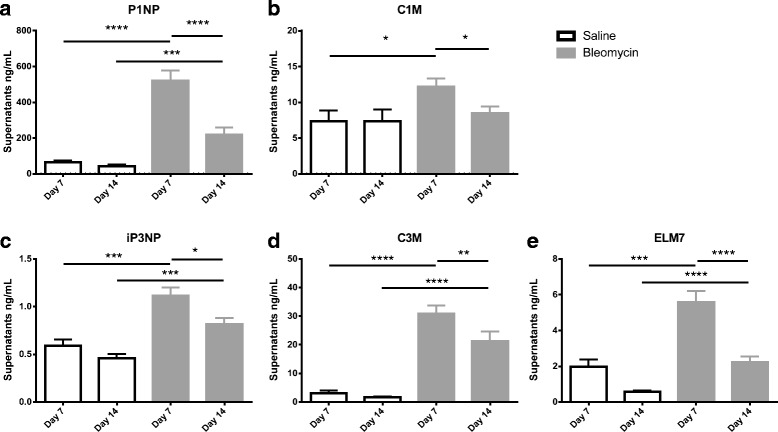
Levels of N-terminal propeptide of collagen type I (**a**), MMP-2, 9 and 13 generated fragments of collagen type I (**b**), N-terminal propeptide of collagen type III (**c**), MMP-9 generated fragments of collagen type III (**d**) and MMP-7 generated fragments of elastin (**e**) in the supernatants of the PCLS model after 48 h of culturing from animals euthanized seven days after last BLM or vehicle dosing and animals euthanized 14 days after the last dosing. w/o: Williams E medium without intervention. Significance level: *p < 0.05, **p < 0.01, *** *p* < 0.001, ****p < 0.0001

There was a 10-fold increase of type I collagen formation (P1NP) in the conditioned medium from BLM animals versus saline control animals at day 7 termination in the PCLS model (*p* < 0.0001) (Fig. 1a). Moreover P1NP was significantly increased in the conditioned medium from BLM animals at day 14 compared to day 14 saline controls (*p* = 0.0008), but significantly decreased when compared to day 7 BLM animals (*p* < 0.0001) (Fig. 1a).

There were increased levels of type I collagen degradation (C1M) in the conditioned medium from BLM animals versus saline control animals in the PCLS model at day 7 termination (*p* = 0.016) (Fig. 1b). In addition, C1M was significantly decreased in the conditioned medium of BLM animals terminated at day 14 when compared to conditioned medium from day 7 termination in the PCLS model (*p* = 0.015) (Fig. 1b).

Type III collagen formation (iP3NP) was significantly increased in the conditioned medium from BLM animals when compared to saline control animals terminated at day 7 in the PCLS model (*p* = 0.0001) (Fig. 1c). The levels of iP3NP were significantly decreased in the conditioned medium from BLM animals terminated at day 14 when compared to the conditioned medium from BLM animals terminated day 7 in the PCLS model (*p* = 0.017), but still significantly elevated compared to day 14 saline controls (*p* = 0.0005) (Fig. 1c).

There were a 15-fold increase in the levels of collagen type III degradation (C3M) in the conditioned medium of BLM animals when compared to saline control animals terminated at day 7 (*p* < 0.0001) in the PCLS model (Fig. 1d). The levels of C3M were significantly decreased in the conditioned medium from BLM animals terminated at day 14 when compared to the conditioned medium from BLM animals terminated at day 7 in the PCLS model (*p* = 0.0008), but still significantly elevated compared to day 14 saline controls (*p* < 0.0001) (Fig. 1d).

Levels of MMP-7 generated elastin fragments (ELM7) were significantly increased the conditioned medium from BLM animals when compared to saline control animals terminated at day 7 (*p* = 0.0002) in the PCLS model (Fig. 1e). The levels of ELM7 were significantly decreased in the conditioned medium from BLM animals terminated at day 14 when compared to the conditioned medium from BLM animals terminated at day 7 (*p* < 0.0001) but still significantly elevated compared to day 14 saline controls (*p* < 0.0001) (Fig. 1e).

### Lung ECM remodeling can be differentially modulated by intervention

**Fig. 2:**
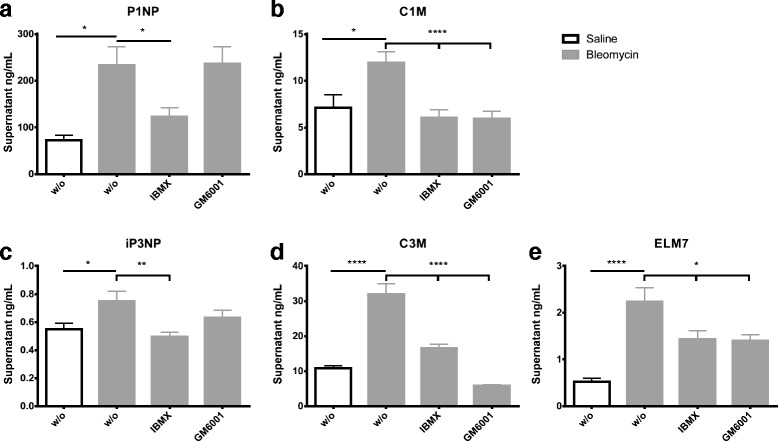
Levels of N-terminal propeptide of collagen type I (**a**), MMP-2, 9 and 13 generated fragments of collagen type I (**b**), N-terminal propeptide of collagen type III (**c**), MMP-9 generated fragments of collagen type III (**d**) and MMP-7 generated fragments of elastin (**e**) in the supernatant of the PCLS model after 48 h of culturing from animals euthanized fourteen days after last BLM or vehicle dosing. w/o: Williams E medium without any intervention; IBMX: Williams E medium + 100 μM IBMX; GM6001: Williams E medium + 10 μM GM6001. Black bars; slices from vehicle rats. Grey bars; slices from BLM-treated rats. Significance level: *p < 0.05, **p < 0.01, ****p < 0.0001

There were significantly increased levels of type I collagen formation (P1NP) in the conditioned medium from BLM animals when compared to saline control animals (*p* = 0.013) in the PCLS model (Fig. 2a). Adding IBMX to the medium significantly decreased the levels (*p* < 0.05), while adding GM6001 had no significant effect (Fig. 2a). There were significantly increased levels of type I collagen degradation (C1M) in the conditioned medium from BLM animals when compared to saline control animals (*p* = 0.014) in the PCLS model (Fig. 2b). Adding IBMX to the medium significantly decreased the levels (*p* < 0.0001), and the same was seen when adding GM6001 (*p* < 0.0001) to the medium (Fig. 2b).

Significantly increased levels of collagen type III formation (iP3NP) were seen in the conditioned medium from BLM animals when compared to saline control animals (*p* < 0.05) in the PCLS model (Fig. 2c). Adding IBMX to the medium significantly decreased the levels (*p* = 0.003), while adding GM6001 had no significant effect (Fig. 2c).

Significantly increased levels of MMP-9 degraded collagen type III (C3M) were observed in the conditioned medium from BLM treated animals when compared to saline control animals (*p* < 0.0001) in the PCLS model (Fig. 2d). There was a 5-fold increase in the supernatant from the BLM treated animals, which could be significantly decreased by adding IBMX (*p* < 0.0001) to the medium, while GM6001 (*p* < 0.0001) was able to decrease the levels below vehicle (Fig. 2d).

Levels of MMP-7 generated fragments of elastin (ELM7) were significantly elevated in the conditioned medium from BLM treated animals when compared to vehicle treated animals (*p* < 0.0001) in the PCLS model (Fig. 2e). It was seen that adding IBMX (*p* = 0.02) and GM6001 (*p* = 0.015) were able to significantly decrease the ELM7 levels.

### Viability was sustained in the PCLS

**Fig. 3:**
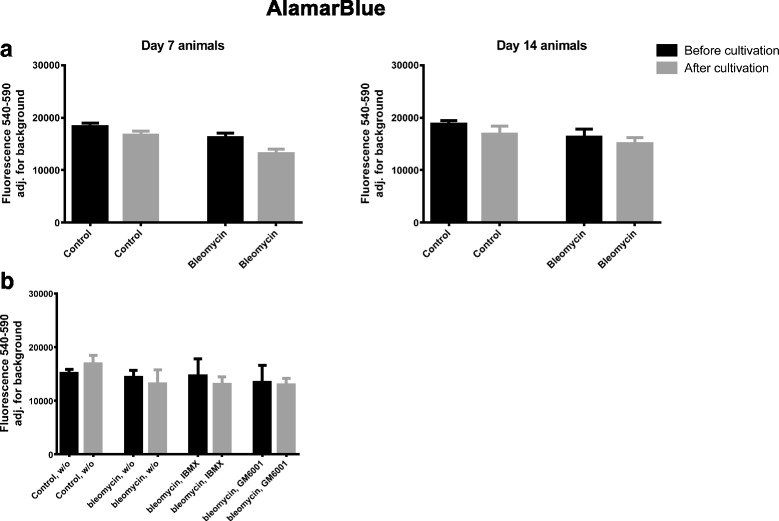
AlamarBlue was investigated as a measure of metabolic activity in fibrotic and control lung slices. **a**) Left graph depicts the metabolic activity in lung slices coming from control and BLM animals euthanized after 7 days, before and after 48 h of cultivation. Right graph depicts the metabolic activity in lung slices coming from control and BLM animals euthanized after 14 days, before and after 48 h of cultivation. **b**) The metabolic activity in lung slices coming from control and BLM animals euthanized after 14 days, before and after 48 h of cultivation. w/o: Williams E medium without any intervention; IBMX: Williams E medium + 100 μM IBMX; GM6001: Williams E medium + 10 μM GM6001

The viability of the PCLS was measured by AlamarBlue at baseline and after 48 h of culturing. Culturing for 48 h or adding IBMX or GM6001 to the lung slices had no effect on metabolic activity (small decrease, not significant) (Fig. 3).

### Histology

**Fig. 4:**
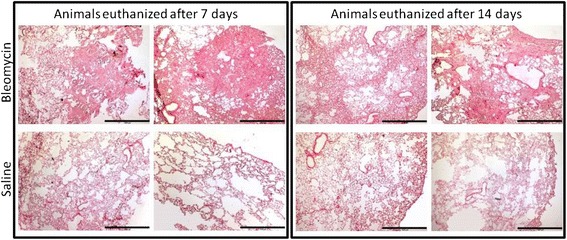
Representative pictures of Sirius Red staining performed on lung tissue from saline control and BLM treated animals. The left section is slides from animals euthanized 7 days after last BLM or saline dose, while the right section is slides from animals euthanized 14 days after last BLM or saline dose. Slides from BML treated animals are depicted in the upper row, while slides from saline treated animals are depicted in the lower row. Pictures were taken at 4x magnification, and the black scale bar in the lower right corner of each picture indicates 1000 μm

**Fig. 5:**
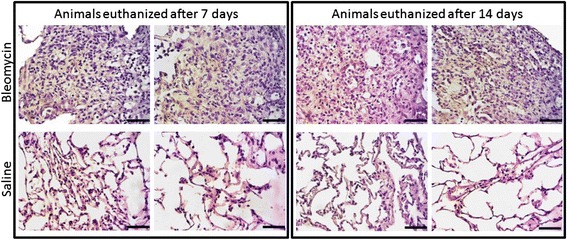
Representative pictures of Orcein Elastin staining performed on lung tissue from saline control and BLM treated animals. Elastin appears brownish. The left section is slides from animals euthanized 7 days after last BLM or saline dose, while the right section is slides from animals euthanized 14 days after last BLM or saline dose. Slides from BML treated animals are depicted in the upper row, while slides from saline treated animals are depicted in the lower row. Pictures were taken at 40x magnification, and the black scale bar in the lower right corner of each picture indicates 100 μm

Sirius Red staining was performed on the lung tissue. It was evident that collagen deposition was present in the lungs of the animals euthanized 7 and 14 days after last BLM dose compared to the saline control animals (Fig. 4). In addition, an Orcein Elastin Stain was performed on the lung tissue, and showed a higher presence of elastin fibers in the lungs of the animals euthanized 7 and 14 days after last BLM dose compared to the saline control animals (Fig. 5). The Orcein stain also clearly shows that the tissue sections coming from BLM rats are more nucleated.

## Discussion

This is, to our knowledge, the first study to evaluate the fibrotic processes in the *in vivo-*like environment of the *ex vivo* PCLS system using novel formation and degradation markers of the ECM to investigate fibrosis remodeling. The animals used for the PCLS model were treated with BLM *in vivo* to induce a disease pattern resembling the IPF seen in human. The PCLS were then cultured in an *ex vivo* setup and the release of formation and degradation epitopes into the supernatant were assessed by ELISA based on monoclonal antibodies.

The highlighted results were: 1) significant differences were seen in the biomarkers levels between saline and BLM treated animals, 2) biomarker levels were inhibited by adding a phosphodiesterase inhibitor or a multiple MMP inhibitor to the culture medium, and 3) levels of the biomarkers decreased with increasing time between last *in vivo* BLM dosing and termination point.

Jenkins et al. recently published a study, in which they evaluated eleven different ECM biomarkers developed by Nordic Bioscience in the PROFILE IPF cohort [2]. Seven of these novel neoepitope biomarkers differed significantly in serum from patients diagnosed with IPF when compared to controls. In addition six of the neoepitope biomarkers were predictive of overall survival of the IPF patients. This paper clearly shows that the ECM remodeling taking place during the pathogenesis of IPF can be assessed by ECM biomarkers. In our rat PCLS model we have chosen ECM remodeling markers equivalent to the markers used by Jenkins et al.

The use of an PCLS model may be an important tool when evaluating ECM diseases such as IPF, since it is well known that cells cultured in traditional monolayer systems differentiate into a non *in vivo*-like phenotype, and may change protein expression profile [19, 26–28]. Another advantage of the PCLS model is the *in vivo-*like pathology kept within this system which may increase the disease relevance. This may improve the success rate when performing drug screening and raise the chance for a new treatment to reach and be implemented in the clinic. In the past decades over 200 drugs have been shown to be effective in the bleomycin model, with only 2 translating into therapeutic agents for IPF. The majority (95 %) of the drugs were given preventive (≤7 days after last BLM dose), likely interfering with the inflammatory and early fibrogenic response [29, 30]. The US Food and Drug Administration (FDA) has stated that “Additional biomarkers and additional surrogate markers are needed to guide product development” [31]. The overall goal of using biomarkers is to improve decision-making with regards to dosing, toxicity and treatment time in order to save costs, which we hope can be applied in the model development.

It is well known that MMPs and other enzymes are upregulated in IPF and play an active role in matrix remodeling [32, 33]. This matrix remodeling can be assessed by biomarkers targeting the neoepitopes generated by MMP cleavage, in both formation and degradation of matrix proteins, thus the markers reflect the tissue balance of the ECM proteins. The results presented here indicate that multiple MMPs are upregulated and involved in the matrix remodeling occurring during disease. It was shown that the system used could be manipulated by adding treatments to the culture medium, suggesting that the model may be used as a system for testing ECM modifying drug candidates. It has previously been reported, within liver and cartilage *ex vivo* setups, that increasing cAMP levels (e.g. by addition of IBMX) affects ECM remodeling [12, 34]. The C3M biomarker has shown to be applicable in a CCl_4_ liver fibrosis *ex vivo* setup. Veidal et al. showed that C3M was significantly elevated in supernatants from CCl_4_ treated animals when compared to vehicle animals. Furthermore by adding IBMX or GM6001 to the culture medium, they saw that the levels of C3M within the supernatants from CCl_4_ treated animals were restored back to the same levels as vehicle treated animals [12]. This indicates that biomarkers are useful in a precision-cut tissue slide (PCTS) setup and that these biomarkers can be inhibited by adding treatments to the culture medium.

It has generally been accepted that collagen type I and III are upregulated in lung tissue of IPF patients [32]. In this paper we show that the formation (P1NP) and degradation (C1M) of type I collagen are significantly increased in supernatants from BLM treated animals, when compared to saline treated animals. In the present study, the levels of both P1NP and C1M could be significantly inhibited by adding IBMX to culture medium, suggesting cAMP has a role in controlling type I collagen expression. The P1NP biomarker is not generated by MMPs, hence the addition of GM6001 did not have an effect on P1NP levels. However, the C1M biomarker is generated from cleavage by MMP-2, -9 and -13, hence adding GM6001 to the culture medium significantly decreased the levels of C1M. Taken together these data suggest an increased turnover of type I collagen in the lungs of animals treated with BLM, as described in the literature but we are the first, as far as we know, to show this turnover in an *ex vivo* culture setup [2, 32, 35].

The levels of type III collagen formation (iP3NP) were significantly elevated in the supernatant of BLM treated animals, while IBMX was able to significantly decrease the levels. Again the iP3NP marker is not generated by MMP cleavage, hence GM6001 had no effect as expected. Our data showed that the degradation of type III collagen (C3M) in supernatants from BLM treated animals was 5-fold higher compared to supernatants from vehicle animals. The levels of C3M were significantly inhibited by adding IBMX or GM6001 to the culture medium. This is in accordance to what was seen in the *ex vivo* liver setup by Veidal et al. These data strongly indicate a higher collagen type III turnover in animals treated with BLM. In the PCLS model we are able to measure upregulated turnover levels of the two most abundant collagens, type I and III. As we are measuring in the supernatants from cultured lung slices, we know that the neo-epitopes are excreted from the lungs, and not subject to background from other parts of the body.

Elastin is a protein involved in the structure, function and elasticity of the ECM in the lungs [36]. MMP-7 is able to degrade elastin and high levels of this enzyme are associated with IPF [37, 38]. ELM7 has previously been reported to be elevated in serum from IPF and lung cancer patients [25]. Our data showed that there are significantly increased levels of ELM7 in the supernatants from BLM treated animals. Adding IBMX could significantly decrease the levels, which confirms that increased cAMP levels can affect the MMP expression as seen by Karsdal et al. and Veidal et al [12, 34]. The ELM7 fragment is MMP generated and adding GM6001 to the culture medium could significantly decrease the levels as expected.

Several studies have shown that the BLM model is known to be reversible with time [29, 39–41], where sacrifice of the animals 14 days after a single I.T. dose of BLM was the most suitable. Chaudhary et al. saw that inflammatory markers are significantly increased during the first 9 days following BLM administration with resolution of the inflammation phase around day 9 [42]. The other interesting observation was the expression of pro-collagen 1 mRNA which begins at day 9 and then decreases back to baseline levels at day 21. These observations lead to the speculation that the switch between inflammation and fibrosis in the rat BLM model occurs around day 9 [42]. Data in this paper shows that the collagen type 1 formation (P1NP) is significantly increased in the supernatants at day 7 following BLM administration when compared to vehicle animals. Interestingly, the levels of P1NP significantly decreases when comparing supernatants at day 14 to day 7 following BLM administration. However, the levels of P1NP in supernatants from BLM treated animals is still significant increased at day 14 following BLM administration when compared to supernatants from vehicle treated animals at day 14. The degradation of type 1 collagen (C1M) was increased in supernatants from BLM treated animals compared to vehicle treated animals at day 7. The levels of C1M in supernatants from BLM animals decreased back to the same levels as saline treated animals at day 14. These data suggest that the type 1 collagen balance is shifted towards deposition of the collagen as observed by Chaudhary et al., but it should be noted that the model described in this paper is a double dose I.T. BLM given two days apart.

Shahzeidi et al. saw that levels of type III procollagen gene expression was increased in mice exposed to single I.T. dose of BLM. Van Hoozen et al. was able to measure elevated levels of type III procollagen mRNA as early as 4 days after administration of a single BLM I.T. dose in a rat model. Razzaque et al. saw a histological increase of type III collagen deposition over a 4 week period following a single BLM I.T. dose in a rat model [43–45]. Our data show that type III collagen formation is significantly elevated 7 days following BLM administration and then it significantly decreases 14 days following BLM administration when compared to day 7. It should be noted that the levels at 14 days following BLM administration are still significantly elevated when compared to vehicle. This goes well with what was seen by Shahzeidi et al. and Van Hoozen et al., but contradicts the histological findings by Razzaque et al.

To our knowledge the turnover of type III collagen in a BLM model has not been described. We saw the same pattern for degradation as with the formation of type III collagen except that there is a 15-fold increase of C3M in supernatants from rats terminated 7 days following BLM administration.

To our knowledge the MMP degradation of ELM7 has not been described in a BLM model. Our findings follow what is seen with the formation of type I and III collagen. The levels are significantly elevated 7 days following BLM administration, which decreases significantly at 14 days following BLM administration when compared to day 7. The levels are still significantly elevated at day 14 when compared to vehicle.

Lastly we show that the lung slices were viable after 48 h of culturing and that the given treatments did not affect the viability. Culturing of 48 h was chosen from previous experiments, showing that viability decreases rapidly after 48 h (data not shown). The histology shows a higher amount of collagen and elastin in the lungs of the animals that were given BLM compared to saline control animals, which correlate well with the data seen in the biomarker analyses.

## Conclusion

We were able to generate a turnover profile in three of the most affected ECM proteins in IPF, collagen type I and III and elastin, in a BLM *ex vivo* model. By adding IBMX and GM6001 we have shown that we were able to significantly decrease the levels of the ECM biomarkers. We then showed that these ECM markers are significantly elevated 7 days following BLM administration and then significantly decreased 14 days after last BLM administration, but still significantly higher than their respective saline controls.

Understanding more on the biology and the interactions of all the cells in the tissue and by giving the treatment after the inflammatory phase, we believe that the risk of having false positives in a murine bleomycin model becomes less. The markers used in the PCLS setup are clinically validated markers shown to be upregulated in serum of IPF patients. This will increase the chances of translation from preclinical species to human, when screening for potential therapies.

## Abbreviations

BLM, Bleomycin; ECM, Extracellular matrix; IBMX, 3-isobutyl-1-methylxanthine; IPF, Idiopathic pulmonary fibrosis; MMP, Matrix metalloproteinase; PCLS, Precision-cut lung slices; PDE, Phosphodiesterase
